# Enabling Solar Water Oxidation by BiVO_4_ in Strongly Acidic Solutions

**DOI:** 10.1021/jacs.5c11785

**Published:** 2025-09-11

**Authors:** Daye Seo, Dae Han Wi, Kyoung-Shin Choi

**Affiliations:** † Department of Chemistry, 5228 University of Wisconsin−Madison, Madison, Wisconsin 53706, United States; ‡ Department of Chemistry, Chungnam National University, Daejeon 34134, Republic of Korea

## Abstract

The oxygen evolution reaction (OER) is paired with various electrochemical and photoelectrochemical reduction reactions used for fuel and chemical production. As there is a strong interest in performing many of these reduction reactions in strongly acidic solutions to increase the reaction rate, efficiency, or selectivity, there is also a great interest in enabling efficient and stable OER in strongly acidic solutions. In this study, we report stable photoelectrochemical OER (POER) of a BiVO_4_ photoanode in 0.1 M HNO_3_ (pH 1). This was achieved by using Nb_2_O_5_ as a protection layer. While Nb_2_O_5_ was rarely used as a protection layer for photoelectrodes in the past, we show its excellent capability to suppress both the chemical and photoelectrochemical dissolution of BiVO_4_ at pH 1. After stabilizing BiVO_4_ with a Nb_2_O_5_ protection layer, we added Co^2+^ ions to the electrolyte as an OER catalyst to enhance the POER. We found that Co_(aq)_^2+^ can serve as a homogeneous OER catalyst without being deposited as a CoO_*x*_ solid catalyst on Nb_2_O_5_. When we performed the POER using unprotected BiVO_4_ with Co_(aq)_^2+^ under the same condition, although POER was enhanced, the enhancement could not be sustained due to the chemical dissolution of BiVO_4_. After the POER, we found that a Co^3+^-containing OER catalyst was deposited on the bare BiVO_4_ surface. This result suggested that the use of Co^2+^ ions as a homogeneous catalyst was possible due to the inertness of the Nb_2_O_5_ surface toward the adsorption or deposition of Co ions. This study enabling stable POER of BiVO_4_ in 0.1 M HNO_3_ using the combination of a Nb_2_O_5_ protection layer and Co_(aq)_^2+^ as a homogeneous OER catalyst provides promising possibilities for acidic POER and OER.

## Graphical abstract

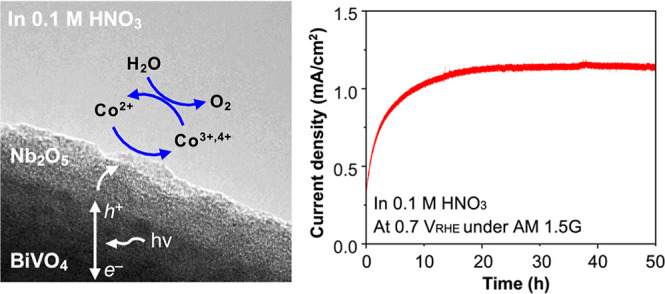

## Introduction

The oxygen evolution reaction (OER) is the most convenient and environmentally benign anode reaction to be coupled with various cathode reactions that produce fuels and chemicals in aqueous media, which include the hydrogen evolution reaction (HER), nitrogen or nitrate reduction, CO_2_ reduction reaction (CO_2_RR), and organic reduction including hydrogenation and hydrogenolysis.^1–4^ There are various advantages to performing many of these cathode reactions in acidic solutions. For example, the kinetics of most HER catalysts are known to be faster in acidic solutions.^5,6^ CO_2_RR in acidic solutions has the advantage of preventing the conversion of CO_2_ to bicarbonate or carbonate, thus allowing for more efficient CO_2_ reduction.^7,8^ Furthermore, recent studies reported that electrochemical hydrodeoxygenation reactions of biomass-derived oxygenates, which are critical to produce biofuels, are enhanced in acidic solutions.^9,10^

When the aforementioned cathode reactions are performed in acidic solutions, the paired OER in the same acidic solutions can create multiple issues. For example, the OER kinetics are reported to be more sluggish in acidic solutions than in alkaline solutions, in contrast to the HER kinetics.^11^ In addition, catalyst stability is another significant challenge for acidic OER; under the combination of strong acidity and anodic bias required for the OER, most nonprecious metal OER catalysts are not stable over long-term use.^12,13^ Thus, there is a critical need for developing inexpensive OER catalysts that are efficient and stable in acidic solutions.

The photoelectrochemical OER (POER) may offer a unique advantage to circumvent the challenges associated with the sluggish kinetics of the OER.^14^ This is because the POER is achieved by holes in the valence band (VB) of a photoanode (i.e., n-type semiconductor), which are generated through photon absorption that excites electrons from the VB to the conduction band (CB). If a photoanode has a valence band maximum (VBM) significantly more positive than 1.23 V vs reversible hydrogen electrode (RHE), the holes at the VBM will have a sufficient overpotential for the OER, without requiring additional electrical bias. Another advantage is that the photoexcited electrons at the conduction band minimum (CBM) of the photoanode will be transferred to the cathode to perform the aforementioned reduction reactions. Since the potential of photoexcited electrons is elevated to that of the CBM of the photoanode, additional electrical bias needed to conduct the desired reduction reaction can be significantly reduced, if the CBM of the photoanode is near or above 0 V vs RHE. Thus, the use of the POER may be particularly beneficial if the operating voltage required to conduct the desired cathode reaction paired with the OER is considerably high (>2 V).

Considering the energetic requirements of the photoanode to meet these criteria, n-type BiVO_4_ is a particularly attractive photoanode. Its VBM is 2.4 V vs RHE, providing more than 1 V of overpotential for the OER, and its CBM is near 0 V vs RHE.^15^ Also, its bandgap (∼2.4 eV) allows for visible light utilization.^15^ However, in order to use BiVO_4_ as a photoanode for the POER in acidic solutions, two critical issues need to be addressed. First, while BiVO_4_ is shown to perform the OER stably in near neutral solutions,^16–18^ BiVO_4_ is not chemically stable in acidic solutions. Thus, BiVO_4_ will require a protection layer to prevent dissolution. Unfortunately, while TiO_2_, the most commonly used protection layer for various photoelectrodes, has been used to protect BiVO_4_ in mildly basic solutions successfully,^19,20^ TiO_2_ as a thin amorphous protection layer appears to be not robust enough to protect BVO_4_ in strongly acidic media, supported by no studies reporting the use of TiO_2_-protected BiVO_4_ in strongly acidic solutions (pH < 2). Thus, the identification of a material that can serve as a more robust protection layer in strongly acidic solutions and a method to deposit this material as a conformal protection layer will be needed. Second, while the holes in BiVO_4_ have sufficient overpotential for the OER, the surface of BiVO_4_ is not catalytic for the OER. Thus, there is still a need to identify stable, efficient, and inexpensive OER catalysts that can best utilize highly oxidizing holes generated in BiVO_4_ for the POER in strongly acidic solutions.

In this study, we present stable POER of BiVO_4_ in 0.1 M HNO_3_ (pH 1), which is enabled by using Nb_2_O_5_ as a protection layer and Co^2+^ as a homogeneous OER catalyst. We report an electrodeposition method to form a conformal coating of Nb_2_O_5_ on a nanoporous BiVO_4_ photoanode having a complex surface morphology and show the capability of Nb_2_O_5_ to protect BiVO_4_ from chemical and photoelectrochemical corrosion. We also show that Co^2+^ ions can serve as a homogeneous OER catalyst on a Nb_2_O_5_-protected BiVO_4_ photoanode, which eliminates not only the need to predeposit the catalyst on Nb_2_O_5_ but also the concerns related to the dissolution loss of the catalyst during the OER in acidic solutions. Our investigation on the unprecedented combination of a Nb_2_O_5_ protection layer and Co^2+^ as a homogeneous OER catalyst offers strategies that may be extended to enhance chemical and photoelectrochemical stabilities of other photoelectrodes.

## Experimental Section

### Materials

Bismuth­(III) nitrate pentahydrate (Bi­(NO_3_)_3_·5H_2_O, 98%), potassium iodide (KI, 99+%), nitric acid (HNO_3_, 70% and 0.1 M), ammonium niobate­(V) oxalate hydrate (NH_4_[NbO­(C_2_O_4_)_2_]·*x*H_2_O, 99.99%), *p*-benzoquinone (*p*-BQ, >98%), vanadium acetylacetonate (VO­(C_5_H_7_O_2_)_2_, 98%), sodium hydroxide (NaOH, >97%), cobalt­(II) nitrate hexahydrate (Co­(NO_3_)_2_·6H_2_O, >98), sodium sulfite (Na_2_SO_3_, >98%), boric acid (H_3_BO_3_, >99.5%) and isopropanol (IPA, 99.9%) were purchased from Sigma-Aldrich. Dimethyl sulfoxide (DMSO, 99.9%) and lactic acid (85%) were purchased from Alfa Aesar Chemicals. Ethanol (200 proof) was purchased from Decon Laboratories. All chemicals were used as purchased without further purification. The deionized water (DI water, >18 MΩ·cm) used in this study was prepared by the Barnstead E-Pure water purification system. Glass substrates coated with fluorine-doped tin oxide (FTO) were purchased from Hartford Glass Co. Nafion membranes (N211) were purchased from Fuel Cell Store.

### Preparation of BiVO_4_/Nb_2_O_5_

Nanoporous BiVO_4_ photoanodes were prepared following the method that our group developed previously,^17^ which uses an electrodeposited BiOI film as a precursor to form a nanoporous BiVO_4_ electrode. The plating solution for BiOI deposition was prepared by mixing four different solutions: (i) 50 mL of an aqueous solution containing 15 mM Bi­(NO_3_)_3_ and 400 mM KI, (ii) 20 mL of ethanol solution containing 46 mM of *p*-BQ, (iii) 169 μL of lactic acid (85%), and (iv) 120 μL of 10-fold-diluted 70% nitric acid. A three-electrode system with an FTO working electrode (WE), an FTO counter electrode (CE), and an Ag/AgCl (4 M KCl) reference electrode (RE) was employed for the electrodeposition. Before use, FTO electrodes were cleaned by sonicating in soapy water and isopropanol successively. For use as the WE, an FTO electrode was masked with PTFE tape such that the exposed area was 1 cm × 1.25 cm. An unmasked FTO electrode, whose size was ∼1.5 × 1.8 cm^2^, was used as the CE. Two different potentials were sequentially applied to the FTO electrodes: (i) −0.33 V vs Ag/AgCl for 20 s to induce Bi nucleation and (ii) −0.1 V vs Ag/AgCl for BiOI growth through *p*-BQ reduction. The detailed deposition mechanisms can be found elsewhere.^17^ The charge passed during the growth step was 0.35 C/cm^2^. As-deposited films were washed with DI water and dried under a stream of air. To convert the BiOI films into n-type BiVO_4_, 65 μL of a DMSO solution containing 200 mM VO­(C_5_H_7_O_2_)_2_ was dropcast onto the BiOI film, and then the film was annealed at 450 °C for 2 h (ramping rate: 2 °C/min) in air. After the thermal treatment, excess V_2_O_5_ was removed by soaking the electrodes in a 1 M NaOH solution for 15 min. BiVO_4_ photoanodes were annealed in N_2_ conditions for another 2 h at 350 °C (ramping rate: 5 °C/min) to further enhance their photocurrent generation.^21^

The Nb_2_O_5_ protection layer was electrochemically deposited on nanoporous BiVO_4_ using the same *p*-BQ reduction. The detailed deposition mechanism is explained below. The electrodeposition used a three-electrode setup composed of a nanoporous BiVO_4_ photoanode as the WE, an FTO CE, and an Ag/AgCl (4 M KCl) RE. The electroplating solution was composed of DMSO and DI water with the 3:2 (v/v) ratio, which contained 10 mM NH_4_[NbO­(C_2_O_4_)_2_]·*x*H_2_O and 100 mM *p*-BQ. The Nb precursor and *p*-BQ were fully dissolved in DMSO first and then DI water was added. The reported concentrations of Nb precursor and *p*-BQ are final concentrations after the DI water is added. The electrodeposition was carried out at −0.6 V vs Ag/AgCl for 10 min. During the deposition, the electrolyte temperature was maintained at 85 °C using an oil bath. The average current density during the deposition process was −2.5 mA/cm^2^.

As-prepared electrodes were washed with ethanol and dried with a gentle steam of air. To dehydrate the NbO_*x*_(OH)_*y*_ layer, thermal treatment was conducted at 350 °C for 2 h (ramping rate: 3.5 °C/min) under a N_2_ environment (flow rate: 10 ccm). These synthesis conditions resulted in the deposition of a 10–15 nm-thick Nb_2_O_5_ layer on BiVO_4_. Deposition of a thinner or thicker Nb_2_O_5_ layer is possible by adjusting the deposition potential or time. For example, a thinner Nb_2_O_5_ layer (e.g., ∼5 nm thick) could be deposited on BiVO_4_ when the electrodeposition potential and time were adjusted to −0.2 V and 5 min (average current density = −1.2 mA/cm^2^), respectively (Figure S1a). However, decreasing the thickness of the Nb_2_O_5_ layer did not result in an increase in photocurrent of the resulting BiVO_4_/Nb_2_O_5_ photoanode (Figure S1b), suggesting that there is no gain for decreasing the thickness of the Nb_2_O_5_ layer below 10–15 nm.

### Materials Characterization

X-ray diffraction (XRD) patterns of the samples were obtained using an X-ray diffractometer (Bruker D8 Discover) with a Cu Kα (λ = 1.54178 Å) radiation source. Scanning electron microscopy (SEM) images of the samples were acquired using a scanning electron microscope (Zeiss Supra VP55). Transmission electron microscopy (TEM) images, high-angle annular dark-field scanning transmission electron microscopy (HAADF-STEM) images and corresponding energy dispersive X-ray spectroscopy (EDS) mapping images were obtained using FEI Tecnai G2 F30 S-TWIN and Talos F200X transmission electron microscope operating at 200 kV. X-ray photoelectron spectroscopy (XPS) spectra were obtained using an X-ray photoelectron spectrometer (Thermo K-Alpha X-ray photoelectron spectrometer) with Al Kα X-ray source. For the calibration of the XPS data, the C 1s peak at 284.8 eV was used as a reference. For the evaluation of chemical stability of BiVO_4_ and BiVO_4_/Nb_2_O_5_ photoanodes, the photoanodes were immersed in 0.1 HNO_3_ (10 mL) for 3 weeks and their stabilities were periodically monitored using SEM and XRD.

### Photoelectrochemical Investigation

All photoelectrochemical measurements were conducted with an SP-200 potentiostat (Bio-Logic Science Instrument). Solar illumination was simulated using an LCS-100 solar simulator (Oriel Instruments) equipped with a 100 W Xe arc lamp (Newport) and an AM 1.5G filter. An infrared filter (Newport) and a focusing lens (Newport) were placed between the light source and the photoelectrode. As back-side illumination was used, the light intensity was calibrated to 100 mW/cm^2^ at the back side of the photoelectrode (i.e., at the FTO surface before the light enters the FTO electrode) using an NREL-certified GaAs reference cell (PV Measurements). The entire area of the BiVO_4_ electrode (∼1.25 cm^2^) was illuminated. A homemade closed quartz cell with a flat window for illumination was utilized. The cells were divided into anodic and cathodic chambers with a glass frit (pore size: 4–8 μm, Ace Glass). Typically, each chamber was filled with 15 mL of solutions for photoelectrochemical measurements. A three-electrode setup composed of a BiVO_4_ photoanode WE, a Pt plate CE, and a Ag/AgCl (4 M KCl) RE was used. The WE and RE were immersed in the anodic chamber while the CE was immersed in the cathodic chamber.

The photoelectrochemical water oxidation was conducted in 0.1 M HNO_3_ (pH 1). When Co_(aq)_^2+^ was utilized as an OER catalyst, 20 mM of Co­(NO_3_)_2_·6H_2_O was added to the solution. The *J*–*V* plots were obtained under chopped illumination to measure the dark current and photocurrent densities during a single potential scan. The potential was scanned from the open circuit potential to the positive direction with a scan rate of 10 mV/s. The *J*–*t* plots were measured at 0.7 V vs RHE. All water oxidation results in this work are presented with respect to RHE for easy comparison with other reports using various pH conditions unless otherwise specified. Potentials versus Ag/AgCl (4 M KCl) reference electrodes (*E*_(vs Ag/AgCl)_) were converted to potentials versus RHE (*E*_(vs RHE)_) using eq 1.

1 $$E_{\left(V\;vs\;RHE\right)}=E_{\left(V\;vs\;Ag/AgCl\right)}+E_{Ag/AgCl\;\left(4\;M\;KCl\right)}+\left(0.0591\;V\times pH\right)$$

where *E*_Ag/AgCl (4 M KCl)_ = 0.1976 V vs SHE at 25 °C.

O_2_ measurement was performed using a gastight closed cell with a Nafion membrane dividing the cathode and anode compartments. The volume of the head space in the anodic chamber was 24 mL and the O_2_ gas was detected using an Ocean Optics fluorescence-based oxygen sensor (Neofox, FOSPOR-R). The probe was inserted into the headspace of the anodic chamber, and the vol % of evolved O_2_ was continuously recorded. The measurement was performed while a constant potential of 0.7 V vs RHE was applied to BiVO_4_/Nb_2_O_5_ (1.25 cm^2^) under illumination. The Faradaic efficiency (FE) was calculated by dividing the amount of gas detected by the theoretical amount of gas calculated on the basis of the total charge passed, using the following equation

2 $$FE\;\left(\%\right)=\frac{4\times n\;\left(mol\right)\times F\;\left(C\;{mol}^{‐1}\right)}{charge\;passed\;\left(C\right)}\times 100\;\left(\%\right)$$

where *n* is moles of evolved O_2_ gas and *F* is the Faraday constant (96,485.33 C mol^–1^).

## Results and Discussion

### Electrodeposition of Nb_2_O_5_ on BiVO_4_

In this study, we used Nb_2_O_5_ as a protection layer to operate high surface area, nanoporous BiVO_4_ photoanodes^16,17^ for POER in strongly acidic solutions. Nb_2_O_5_ was chosen as it is one of the few oxides that are chemically stable in strongly acidic solutions across a wide potential range,^22^ but has rarely been explored as a protection layer for semiconductor photoelectrodes.^23^ In order to form a thin conformal Nb_2_O_5_ layer on BiVO_4_, we electrodeposited Nb_2_O_5_ using the nanoporous BiVO_4_ electrode as the WE.

The water-soluble NH_4_[NbO­(C_2_O_4_)_2_]·*x*H_2_O was used as the Nb precursor, and the electrodeposition of Nb_2_O_5_ was achieved using the reduction of *p*-BQ to hydroquinone (eq 3). This reduction reaction consumes protons and increases the local pH on the BiVO_4_ surface, which decreases the solubility of the Nb^5+^ precursor, resulting in the precipitation of Nb^5+^ as NbO_*x*_(OH)_*y*_, the hydrated form of Nb_2_O_5_, coating the BiVO_4_ surface. While there are other reduction reactions (e.g., HER and nitrate reduction) that can consume H^+^ or generate OH^–^ to increase the local pH on the WE,^24^ they are not viable when BiVO_4_ or Bi^3+^ containing material is used as the WE. This is because the thermodynamic potential and kinetic overpotential needed for these reduction reactions can reduce Bi^3+^ in BiVO_4_, destroying the BiVO_4_ electrode.^19,25^ In contrast, the reduction of *p*-BQ can be achieved without changing the composition of the BiVO_4_ WE. After the deposition, the NbO_*x*_(OH)_*y*_ layer was dehydrated to Nb_2_O_5_ by annealing at a mild condition (350 °C with N_2_ for 2 h), resulting in the formation of Nb_2_O_5_-coated BiVO_4_, BiVO_4_/Nb_2_O_5_.


3

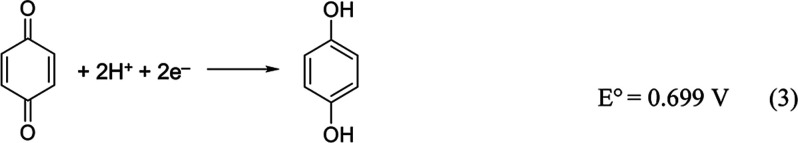



The SEM images of BiVO_4_ and BiVO_4_/Nb_2_O_5_ showed no noticeable difference (Figure 1a,b), confirming that the nanoporous structure of BiVO_4_ remained intact during the electrodeposition of Nb_2_O_5_. Also, they suggested that Nb_2_O_5_ was deposited as a thin layer with no distinguishable morphology. The presence of a thin, conformal Nb_2_O_5_ layer on BiVO_4_ was verified using multiple methods. First, Nb 3d XPS spectra showed the presence of Nb^5+^ on the BiVO_4_/Nb_2_O_5_ surface; the two characteristic peaks at 207.5 and 210.3 eV correspond to 3d_5/2_ and 3d_3/2_ peaks of Nb^5+^ (Figure 1c). Second, the TEM image of BiVO_4_/Nb_2_O_5_ showed that the Nb_2_O_5_ layer was 10–15 nm thick (Figure 1d). The fast Fourier transform (FFT) of the Nb_2_O_5_ region in the bright-field image revealed that the Nb_2_O_5_ layer was amorphous (Figure 1d, inset), consistent with the XRD pattern of BiVO_4_/Nb_2_O_5_ which did not show any Bragg peaks corresponding to crystalline Nb_2_O_5_ (Figure S2). Lastly, the HAADF-STEM-EDS mapping additionally confirmed that the Nb_2_O_5_ layer uniformly covered the BiVO_4_ surface with no notable pinholes in the numerous specimens we examined (Figure 1e).

**Figure 1:**
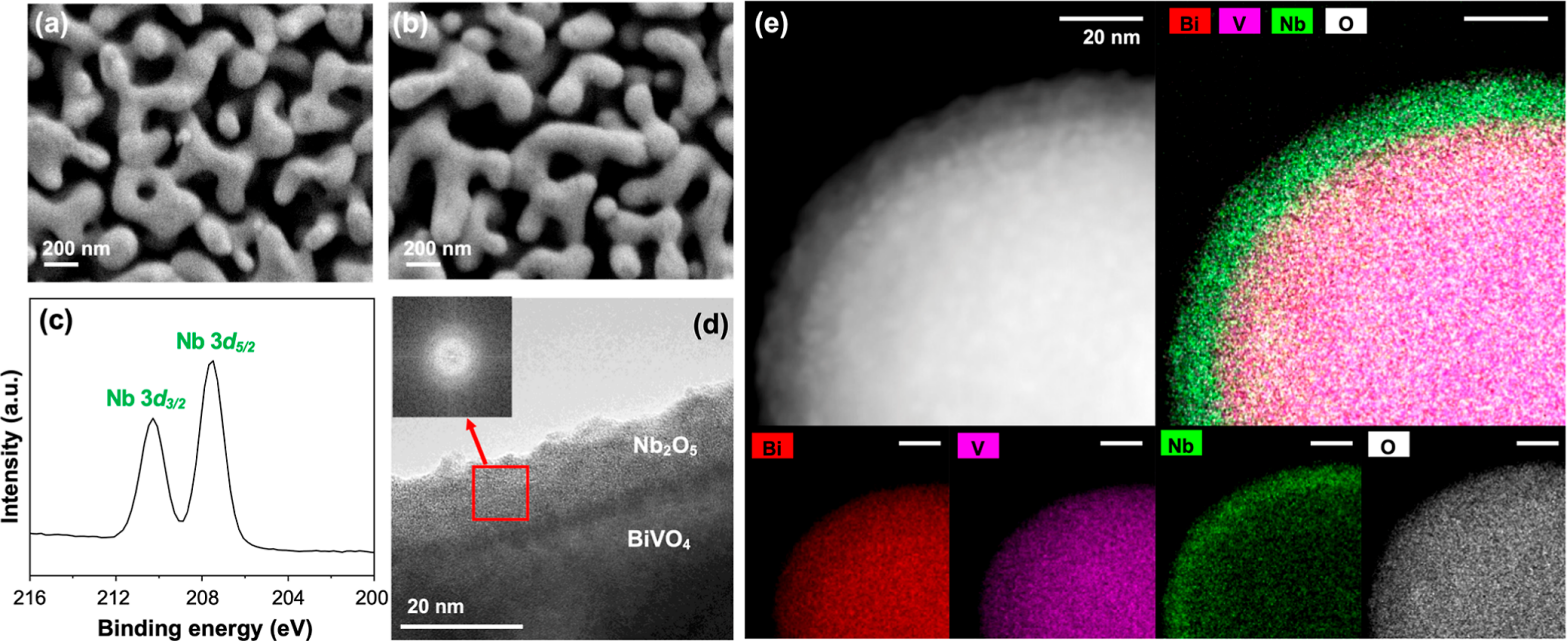
Characterization of BiVO_4_/Nb_2_O_5_. SEM images of (a) bare BiVO_4_ and (b) BiVO_4_/Nb_2_O_5_ photoanodes. (c) Nb 3d XPS spectrum of BiVO_4_/Nb_2_O_5_. (d) A representative TEM image of BiVO_4_/Nb_2_O_5_ with an inset showing the FFT of the Nb_2_O_5_ region. (e) HAADF-STEM image and corresponding EDS element mapping images of BiVO_4_/Nb_2_O_5_. All scale bars in (e) are 20 nm.

### Chemical Stability of BiVO_4_/Nb_2_O_5_ at pH 1

Before evaluating the effect of Nb_2_O_5_ on the photoelectrochemical property and stability of BiVO_4_, we first examined the ability of Nb_2_O_5_ to protect BiVO_4_ from chemical dissolution in 0.1 M HNO_3_ (pH 1) for 21 days of immersion. As unprotected BiVO_4_ would dissolve in this solution, this test allows us to examine two things. First, a 10–15 nm thick, amorphous Nb_2_O_5_ layer is stable and robust enough in 0.1 M HNO_3_ to protect BiVO_4_ from dissolution. Second, the Nb_2_O_5_ layer is pinhole-free. If pinholes, not detected by the TEM study, indeed exist in the Nb_2_O_5_ layer, BiVO_4_ should dissolve gradually during the 3 weeks of the immersion time. We believe that this type of long-term immersion test offers the most rigorous method to verify the uniformity, conformality, and pinhole-free nature of the Nb_2_O_5_ layer.

Figure 2 shows the comparison of XRD and SEM results of BiVO_4_ and BiVO_4_/Nb_2_O_5_ obtained before and after 7 and 21 days of immersion in 0.1 M HNO_3_. Not surprisingly, the loss of BiVO_4_ by dissolution was evident after 7 days of immersion; the XRD results show a decrease in the BiVO_4_ peaks and SEM images show the loss of BiVO_4_. For the case of BiVO_4_/Nb_2_O_5_, no noticeable changes in SEM images and XRD patterns were observed even after 21 days of immersion, confirming the absence of pinholes in the electrodeposited Nb_2_O_5_ layer as well as the excellent stability of the electrodeposited Nb_2_O_5_ coating on BiVO_4_.

**Figure 2:**
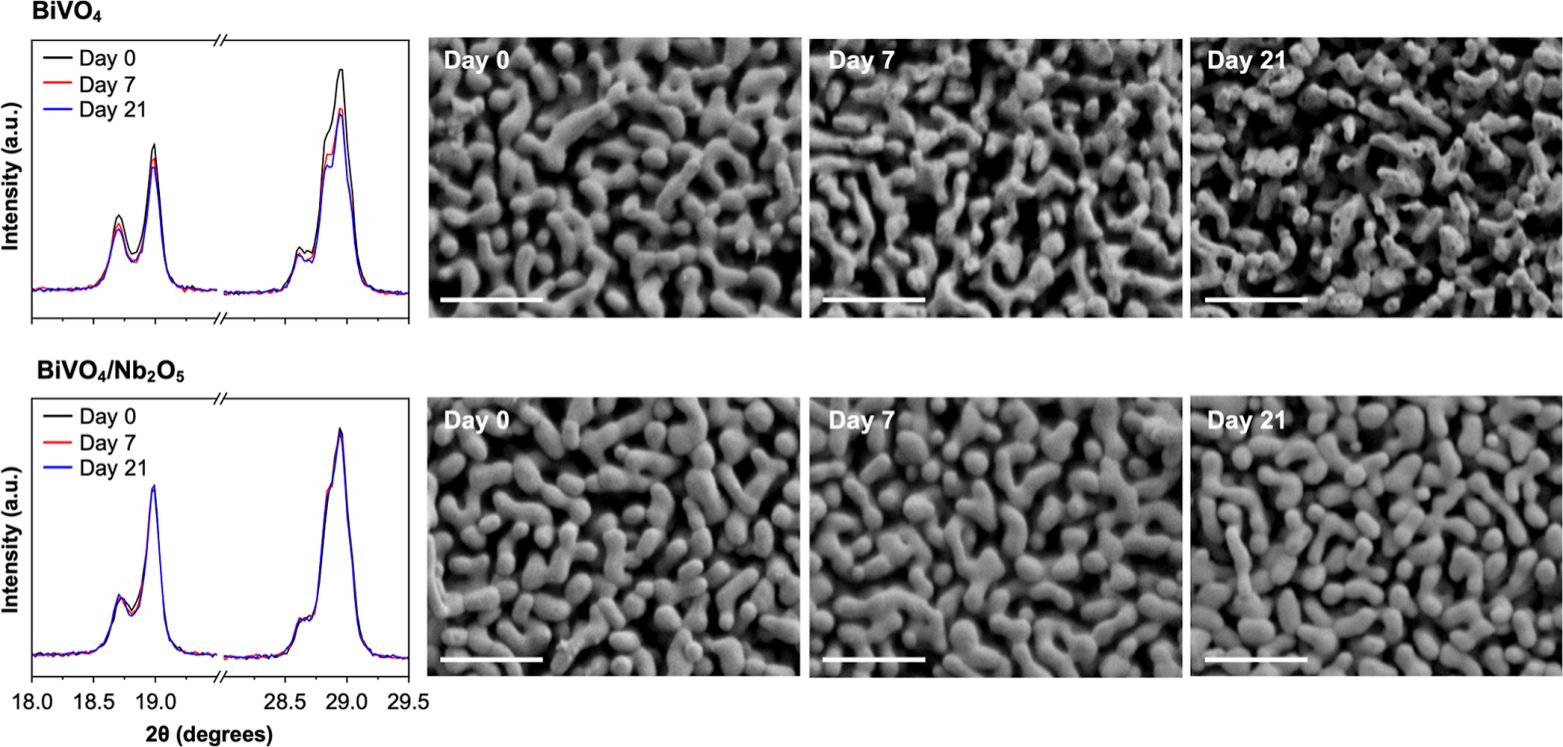
Effect of the Nb_2_O_5_ layer on the chemical stability of BiVO_4_ in 0.1 M HNO_3_ (pH 1). XRD peaks (left) and SEM images (right) of bare BiVO_4_ (top) and BiVO_4_/Nb_2_O_5_ (bottom) measured before and after 7 and 21 days of immersion. Scale bars in the SEM images are 1 μm.

### Photoelectrochemical Properties of BiVO_4_/Nb_2_O_5_

Next, we compared the photoelectrochemical properties and stabilities of BiVO_4_ and BiVO_4_/Nb_2_O_5_ in 0.1 M HNO_3_ under AM 1.5G illumination. It should be noted that protecting BiVO_4_ against anodic photocorrosion is much more challenging than protecting BiVO_4_ against chemical dissolution in the same acidic solution. This is because while the chemical dissolution of BiVO_4_ caused by acid can be prevented by blocking the direct contact between BiVO_4_ and acid by placing an acid-stable protection layer, the anodic photocorrosion of BiVO_4_ is induced by surface-reaching photogenerated holes within the BiVO_4_ underneath the protection layer. When the surface-reaching holes are not quickly consumed for water oxidation, the accumulated holes on the surface can oxidize Bi^3+^ or O^2–^ in the BiVO_4_ lattice. This anodic photocorrosion destroys the BiVO_4_ surface and weakens the BiVO_4_/protection layer interface, allowing the acidic solution to reach the surface of BiVO_4_, further accelerating the dissolution of BiVO_4_ by chemical and photoelectrochemical corrosion. Thus, a protection layer that can prevent the acidic dissolution can still fail to prevent anodic photocorrosion. In order for the protection layer to suppress photocorrosion, its composition and structure should be extremely robust such that it can hinder the interfacial atomic rearrangement on the BiVO_4_ surface that needs to be coupled for the photooxidation of BiVO_4_. If the interfacial atomic rearrangement is difficult to occur, it can effectively slow down the rate of photocorrosion relative to the rates of surface recombination and the OER, allowing the surface holes to be consumed for surface recombination and OER instead, thereby preventing photocorrosion.

The comparison of the *J*–*V* plots of BiVO_4_ and BiVO_4_/Nb_2_O_5_ photoanodes for the POER in 0.1 M HNO_3_ is shown in Figure 3a. BiVO_4_ showed higher photocurrent generation than BiVO_4_/Nb_2_O_5_, suggesting that the presence of Nb_2_O_5_ hindered efficient hole transfer. However, the advantage of the Nb_2_O_5_ layer was revealed by the *J*–*t* measurements. The *J*–*t* plot of BiVO_4_ at 0.7 V vs RHE showed that the photocurrent of BiVO_4_ increased gradually for the initial 2 h and then decreased to around 0.4 mA/cm^2^ at 12 h (Figure 3b). This behavior suggests that the initial photocurrent of BiVO_4_ was partly from photocorrosion, and that the initial photocurrent increase was due to the increase in surface area of BiVO_4_ caused by photocorrosion-induced dissolution of BiVO_4_. As more dissolution of BiVO_4_ occurred, it inherently decreased photon absorption by BiVO_4_. Also, due to numerous recombination sites generated on the disintegrated BiVO_4_ surface, an increasing fraction of surface-reaching holes was lost to surface recombination. As a result, the photocurrent of BiVO_4_ could not be sustained and decreased over time. Indeed, the postanalysis of BiVO_4_ using XRD and SEM after the 12 h-long *J*–*t* plot measurement showed a considerable loss of BiVO_4_ by its anodic photocorrosion (Figure 3c,d).

**Figure 3:**
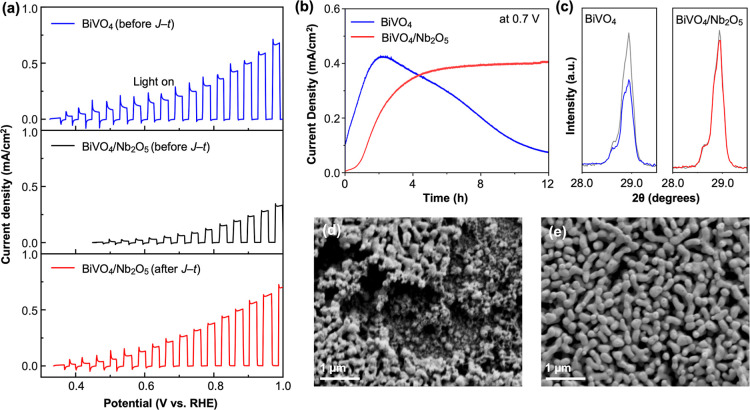
Effect of the Nb_2_O_5_ layer on the photoelectrochemical stability of BiVO_4_ at pH 1. (a) *J*–*V* plots of BiVO_4_ (blue) and BiVO_4_/Nb_2_O_5_ (black and red) measured in 0.1 M HNO_3_ under AM 1.5G illumination (scan rate: 10 m V/s). (b) *J*–*t* plots of bare BiVO_4_ (blue) and BiVO_4_/Nb_2_O_5_ (red) at 0.7 V vs RHE. (c) XRD peak intensity change of BiVO_4_ (blue) and BiVO_4_/Nb_2_O_5_ (red) after the *J*–*t* measurement. The most intense (112) peak of BiVO_4_ is shown as an example peak, and the gray line shows the peak of the pristine BiVO_4_. SEM images of (d) BiVO_4_ and (e) BiVO_4_/Nb_2_O_5_ after the *J*–*t* measurement.

In contrast, BiVO_4_/Nb_2_O_5_ showed a gradual increase in photocurrent during the first 4 h but the increased photocurrent was stably maintained for the next 8 h (Figure 3b). The postanalysis of BiVO_4_/Nb_2_O_5_ using XRD after the 12 h-long *J*–*t* measurement showed no notable changes in the XRD peak intensity of BiVO_4_ (Figure 3c). Also, the SEM image of BiVO_4_/Nb_2_O_5_ after the *J*–*t* measurement shows no change in the photoanode morphology (Figure 3e). These results confirm that Nb_2_O_5_ can effectively suppress anodic photocorrosion of BiVO_4_. These results also mean that the initial photocurrent increase was not due to photocorrosion but due to some activation process of the Nb_2_O_5_ layer, which helped enhance POER of BiVO_4_/Nb_2_O_5_. The enhanced POER of BiVO_4_/Nb_2_O_5_ after the *J*–*t* measurement was also shown in the comparison of *J*–*V* plots before and after the *J*–*t* measurement (Figure 3a).

To better understand the effects of this activation process, we also compared photocurrents for sulfite oxidation and dark OER performances before and after the 12 h-long *J*–*t* measurement (Figure S3). Sulfite is an excellent hole scavenger and all surface-reaching holes can contribute to photocurrent generation for sulfite oxidation.^24^ Thus, comparing photocurrents for sulfite oxidation can reveal the effect of the activation process on the number of surface-reaching holes. On the other hand, comparing dark OER performances can reveal the effect of the activation process on the OER catalytic ability. Our results showed that the activation process helped improve both the number of holes reaching the surface and the OER catalytic ability (Figure S3a,b). However, no changes in the crystallinity and oxidation state of Nb_2_O_5_ were detected by TEM and XPS analyses after the activation process (Figure S3c,d), which means that the improvements by the activation process were achieved through subtle atomic rearrangements at the BiVO_4_/Nb_2_O_5_ junction, within the Nb_2_O_5_ layer, and on the Nb_2_O_5_ surface without involving long-range ordering or composition change in Nb_2_O_5_.

We note that the results of BiVO_4_/Nb_2_O_5_ in Figure 3 are particularly remarkable since no OER catalyst was used in this experiment. Without a proper OER catalyst, more holes can accumulate at the surface, increasing the rate of photocorrosion. The fact that Nb_2_O_5_ was able to suppress photocorrosion of BiVO_4_, even when an OER catalyst was not present, suggests that Nb_2_O_5_ offers excellent structural rigidity at the BiVO_4_/Nb_2_O_5_ interface, making the surface atomic reorganization of BiVO_4_ needed for photocorrosion extremely difficult. This makes the rate of photocorrosion slower than the rates of the OER and surface recombination, thus effectively suppressing the photocorrosion of BiVO_4_. The *J*–*V* plot of BiVO_4_/Nb_2_O_5_ was measured again after the *J*–*t* measurement (Figure 3a, red), and it showed a significantly enhanced photocurrent compared to the initial *J*–*V* plot (Figure 3a, black) because BiVO_4_/Nb_2_O_5_ was fully activated during the *J*–*t* measurement. In contrast, the *J*–*V* plot of BiVO_4_ measured again after the *J*–*t* measurement showed negligible photocurrent (Figure S4).

### POER of BiVO_4_/Nb_2_O_5_ with Co^2+^ as an OER Catalyst

Next, we combine the BiVO_4_/Nb_2_O_5_ photoanode with an OER catalyst so that a greater portion of the surface-reaching holes can be used for the POER instead of surface recombination. Among various non-noble metal-based OER electrocatalysts, Co-based catalysts have shown notable catalytic activities in acidic solutions.^12,26,27^ However, despite many improvements, the dissolution loss of Co remains a significant concern.^12^

In our experiment, the POER of BiVO_4_/Nb_2_O_5_ was performed in 0.1 M HNO_3_ containing 20 mM Co^2+^ without predepositing a Co-containing OER catalyst on Nb_2_O_5_. It is known that Co_(aq)_^2+^ in solutions can be oxidized to less soluble Co^3+^ or Co^4+^ under anodic bias used for the OER and it can be deposited as a solid OER catalyst on the electrode surface during the OER.^28,29^ Even if the Co catalyst is reduced back to Co^2+^ during the OER catalytic cycle and dissolves, the resulting Co_(aq)_^2+^ can be reoxidized, regenerating the solid catalyst and achieving stable OER performance.^29^ Thus, we investigated how Co_(aq)_^2+^ can utilize holes from BiVO_4_/Nb_2_O_5_ for the OER in 0.1 M HNO_3_.

The *J*–*t* plots of BiVO_4_/Nb_2_O_5_ obtained at 0.7 V vs RHE with and without Co^2+^ show that the presence of Co^2+^ increased photocurrent generation significantly. For example, at 12 h, the photocurrent density achieved with Co^2+^ was 0.97 mA/cm^2^, which is around 2.5 times increase from 0.41 mA/cm^2^ achieved without Co^2+^ (Figure 4a). To verify that the photocurrent generated in the presence of Co^2+^ was indeed associated with the OER, we quantified photoelectrochemically produced O_2_ during the *J*–*t* measurement and the Faradaic efficiency for the OER was calculated to be >90% (Figure 4c). The *J*–*V* plots obtained with and without Co^2+^ after the *J*–*t* measurements also clearly show a remarkable photocurrent increase caused by the presence of Co^2+^ over the entire potential region (Figure 4b).

**Figure 4:**
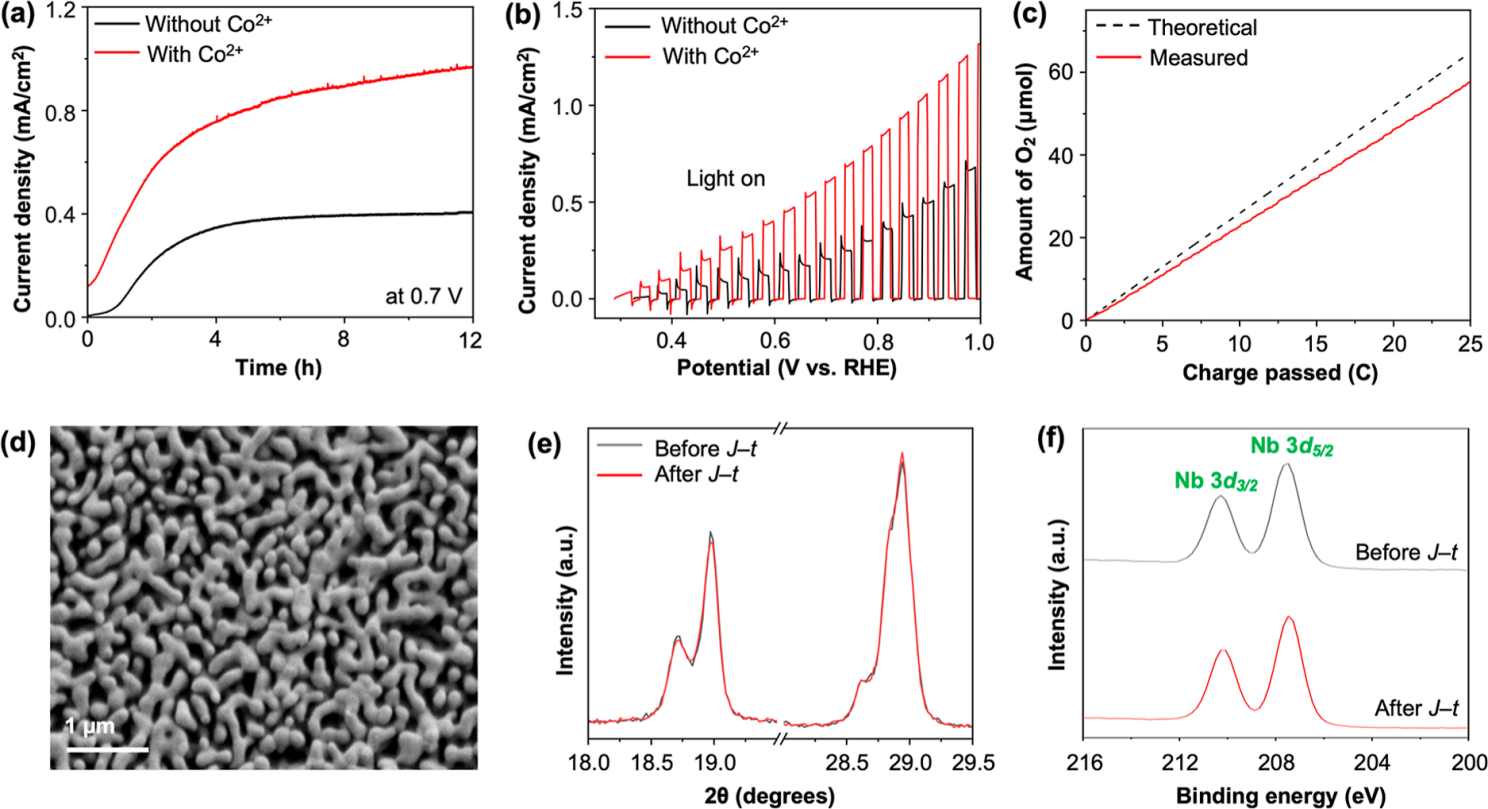
POER of BiVO_4_/Nb_2_O_5_ with 20 mM Co^2+^. (a) *J*–*t* plots at 0.7 V vs RHE and (b) *J*–*V* plots of BiVO_4_/Nb_2_O_5_ obtained in 0.1 M HNO_3_ under AM 1.5G illumination (100 mW/cm^2^) with (red) and without (black) Co^2+^. The *J*–*V* plots were measured after the *J*–*t* plot measurements shown in (a). (c) Theoretical (black dotted line) and detected (red solid line) O_2_ produced during the *J*–*t* measurement with Co^2+^ at 0.7 V vs RHE. (d) SEM image, (e) XRD peaks, and (f) Nb 3d XPS spectra of BiVO_4_/Nb_2_O_5_ after the *J*–*t* measurement with Co^2+^. Gray traces in (e,f) are measured before the *J*–*t* measurement.

After the *J*–*t* measurement, the BiVO_4_/Nb_2_O_5_ photoanode was examined by SEM, XRD, and XPS, and no noticeable changes in the surface morphology, crystallinity, and quantity of BiVO_4_ as well as the presence of Nb_2_O_5_ were detected (Figure 4d–f). An interesting discovery from the post *J*–*t* analysis of BiVO_4_/Nb_2_O_5_ is that there was no indication that Co^2+^ was deposited as a solid OER catalyst (i.e., CoO_*x*_) on BiVO_4_/Nb_2_O_5_ during the POER. The presence of Co^2+^ on the BiVO_4_/Nb_2_O_5_ surface was not detected even by XPS, although XPS is very sensitive to the surface composition (Figure 5a), implying that Co^2+^ in our system served as a homogeneous OER catalyst rather than a heterogeneous catalyst.

**Figure 5:**
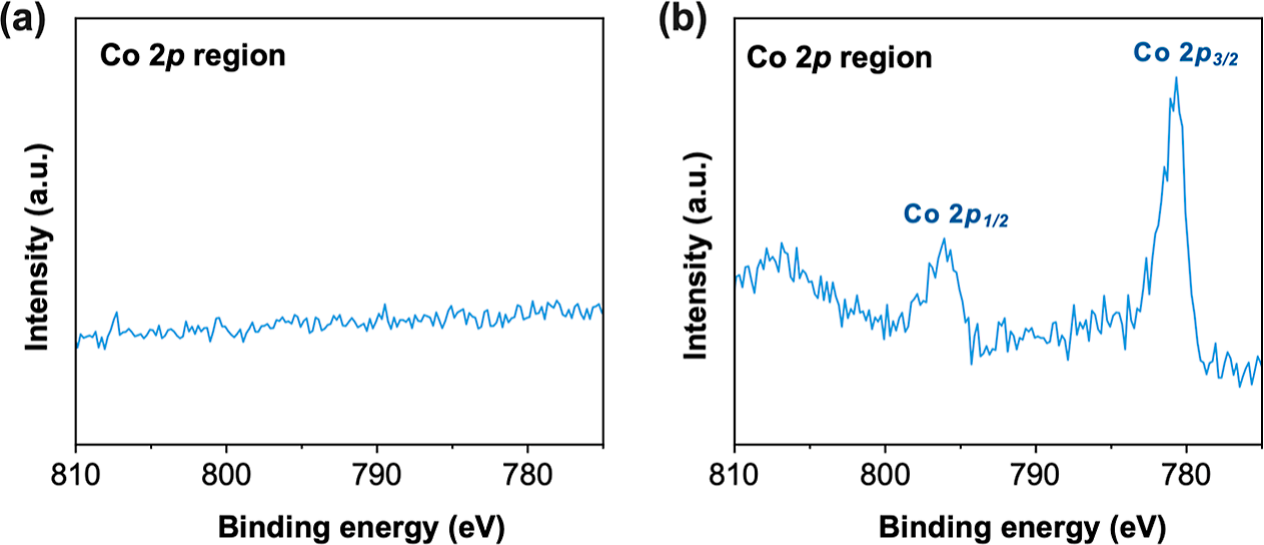
Presence of a CoO_*x*_ catalyst on Nb_2_O_5_ after POER. Co 2p XPS spectra of (a) BiVO_4_/Nb_2_O_5_ and (b) BiVO_4_ obtained after the 12 h *J*–*t* measurement in the presence of Co^2+^.

We also performed a control experiment where the POER of unprotected BiVO_4_ was performed with Co_(aq)_^2+^. The *J*–*V* and *J*–*t* plots of BiVO_4_ with Co^2+^ showed a considerable increase in photocurrent generation when Co^2+^ was present, as Co^2+^ helped BiVO_4_ to consume a higher portion of surface-reaching holes for the POER (Figure 6a,b), which should decrease the portion of surface-reaching holes consumed for photocorrosion. Indeed, the XRD (Figure 6c) and SEM results (Figure S5) of unprotected BiVO_4_ after the *J*–*t* measurement with Co^2+^ showed that the photocorrosion induced-dissolution of BiVO_4_ was decreased notably compared to the case without Co^2+^. However, Co^2+^ alone could not protect the inherent chemical dissolution of BiVO_4_ in a strongly acidic solution, and the post *J*–*t* analysis showed evident destruction of BiVO_4_, unlike the case of BiVO_4_/Nb_2_O_5_. This explains why the POER of BiVO_4_ with Co^2+^ could not be sustained, resulting in a gradual decrease in the photocurrent over time (Figure 6b). An interesting result to note is that when unprotected BiVO_4_ was used with Co_(aq)_^2+^ for the POER, the post *J*–*t* XPS analysis of BiVO_4_ showed a significant amount of Co^3+^ present on the BiVO_4_ surface (Figure 5b). This means that Co_(aq)_^2+^ serving as a homogeneous OER catalyst is unique to the BiVO_4_/Nb_2_O_5_ surface, and that the Nb_2_O_5_ surface is particularly inert toward the deposition of CoO_*x*_ as a solid catalyst on its surface.

**Figure 6:**
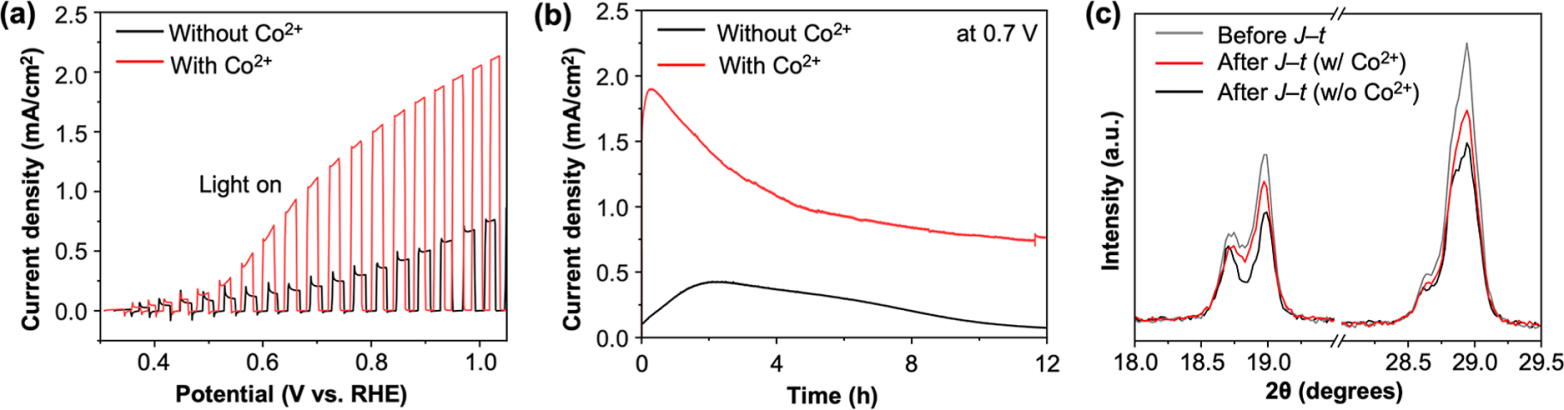
Effect of Co^2+^ on the photoelectrochemical stability of bare BiVO_4_ photoanodes at pH 1. (a) *J*–*V* plot and (b) *J*–*t* plot at 0.7 V vs RHE of bare BiVO_4_ in 0.1 M HNO_3_ under AM 1.5G illumination (100 mW/cm^2^) with (red) and without (black) Co^2+^. (c) XRD peaks of BiVO_4_ after the *J*–*t* measurement. Gray trace shows the XRD peaks of pristine BiVO_4_.

Finally, we examined the longer-term stability of BiVO_4_/Nb_2_O_5_ with Co^2+^ over 50 h in 0.1 M HNO_3_. After an initial activation period, the photocurrent density of BiVO_4_/Nb_2_O_5_ reached to 1.15 mA/cm^2^, which was maintained stably for the remaining time (Figure 7a). The SEM image and XRD result of BiVO_4_/Nb_2_O_5_ after 50 h of *J*–*t* measurements still did not show any indication of chemical or photoelectrochemical degradation of BiVO_4_ (Figure 7b,c). This level of stable POER operation of oxide-based photoanodes that are chemically unstable in strongly acidic solutions has not been demonstrated previously,^30,31^ which testifies a remarkable protection capability of an electrodeposited Nb_2_O_5_ thin layer along with its unique ability to use Co^2+^ as a homogeneous OER catalyst.

**Figure 7:**
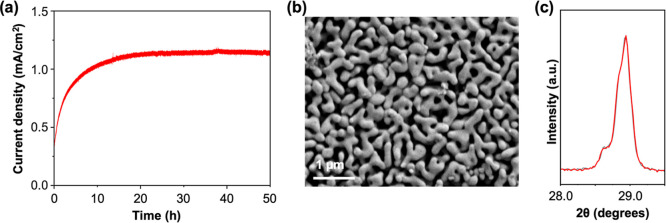
A longer-term stability of BiVO_4_/Nb_2_O_5_ for POER with Co^2+^ at pH 1. (a) *J*–*t* measurement at 0.7 V vs RHE in 0.1 M HNO_3_ under AM 1.5G illumination (100 mW/cm^2^). (b) SEM image and (c) XRD result of BiVO_4_/Nb_2_O_5_ after the *J*–*t* measurement. Gray trace shows the XRD peak of pristine BiVO_4_.

## Conclusions

In this study, we demonstrated a conformal coating of Nb_2_O_5_ on a morphologically complex nanoporous BiVO_4_ photoanode using electrodeposition to use the resulting Nb_2_O_5_ layer as a protection layer to operate BiVO_4_ photoanodes in a strongly acidic solution. The resulting BiVO_4_/Nb_2_O_5_ electrode showed an excellent resistance against chemical dissolution of BiVO_4_ in 0.1 M HNO_3_ (pH 1) for 3 weeks, which confirmed the uniformity and pinhole-free nature of the electrodeposited Nb_2_O_5_ layer as well as the chemical stability of a thin, amorphous Nb_2_O_5_ layer in 0.1 M HNO_3_. When the BiVO_4_/Nb_2_O_5_ photoanode was tested for POER, the presence of Nb_2_O_5_ suppressed anodic photocorrosion of BiVO_4_ even when no OER catalyst was used and Nb_2_O_5_ is not particularly catalytic for OER. This result revealed remarkable chemical and structural robustness of Nb_2_O_5_, not allowing for atomic reorganization at the BiVO_4_/Nb_2_O_5_ interface that needs to be coupled for the holes to photo-oxidize the BiVO_4_ surface. This effectively decreased the rate of photocorrosion compared to the rates of OER and surface electron–hole recombination, thus kinetically preventing the consumption of surface holes for photocorrosion. When we added Co^2+^ ions to the electrolyte, Co_(aq)_^2+^ served as a homogeneous OER catalyst and enhanced the POER of BiVO_4_/Nb_2_O_5_. The use of Co_(aq)_^2+^ as a homogeneous OER catalyst eliminated the need to predeposit an OER catalyst on Nb_2_O_5_ as well as the concern of losing the heterogeneous OER catalyst during acidic POER due to catalyst instability. We found that when unprotected BiVO_4_ was used with Co_(aq)_^2+^, Co^2+^ was deposited as a Co^3+^-containing catalyst on BiVO_4_, suggesting that the use of Co_(aq)_^2+^ as a homogeneous OER catalyst is unique on the Nb_2_O_5_ surface due to the inertness of the Nb_2_O_5_ surface for CoO_*x*_ deposition. This study, which demonstrated stable POER of BiVO_4_ at pH 1, revealed multiple advantages of using Nb_2_O_5_ as a protection layer, which may be used to protect other photoelectrodes.

## Supplementary Material



## Notes

Results obtained with a thinner (∼5 nm thick) Nb_2_O_5_ layer, the XRD pattern of BiVO_4_/Nb_2_O_5_, results showing the effect of the activation process of Nb_2_O_5_ on photoelectrochemical sulfite oxidation and OER in the dark, additional results showing the performances of unprotected BiVO_4_ (PDF)
