# Supplementary material for: Enabling Solar Water Oxidation by BiVO4 in Strongly Acidic Solutions
Source: J Am Chem Soc. 2025 Sep 11;147(38):35002–10. doi: 10.1021/jacs.5c11785 (PMC12503356; doi:10.1021/jacs.5c11785)
Supplement: Supplementary file 1 [file ja5c11785_si_001.pdf]

Supporting Information

Enabling Solar Water Oxidation by $BiVO_4$ in Strongly Acidic Solutions

Daye Seo,[1,†] Dae Han Wi,[1,2†] and Kyoung-Shin Choi[1,*]

[1]*Department of Chemistry, University of Wisconsin-Madison, Madison, WI 53706, United States*

[2]*Department of Chemistry, Chungnam National University, Daejeon 34134, Republic of Korea*

[†] These authors contributed equally to this work.

* Correspondence and requests for materials should be addressed to K.-S.C.
(email: kschoi@chem.wisc.edu).

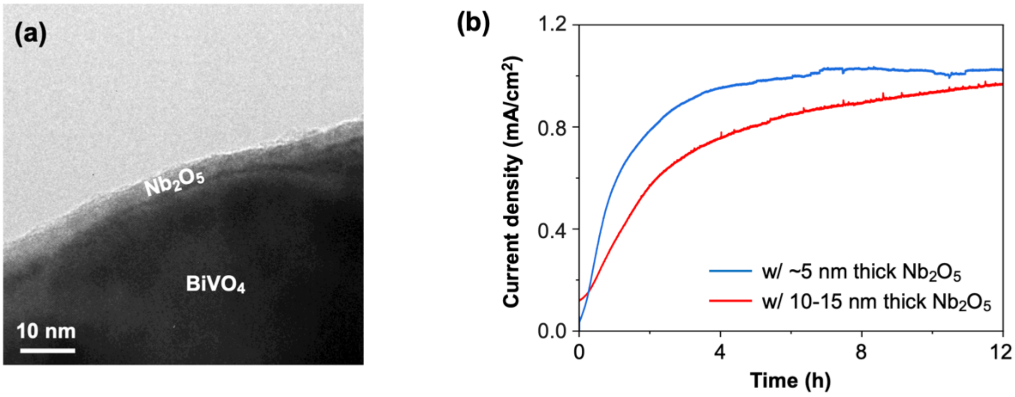

Figure S1. (a) TEM image of $BiVO_4$ coated with a ~5 nm thick Nb_2O_5 layer. (b) The *J–t* plots of $BiVO_4/Nb_2O_5$ samples, one with a ~ 5 nm thick Nb_2O_5 layer (blue) and the other with 10-15 nm thick Nb_2O_5 layer (red), in 0.1 M HNO_3 containing 20 mM Co^{2+} under AM 1.5G illumination (100 mW/cm^2). The applied potential was 0.7 V vs RHE. The thin Nb_2O_5 layer needed less activation time, but the photocurrent densities of the two samples became comparable over time.

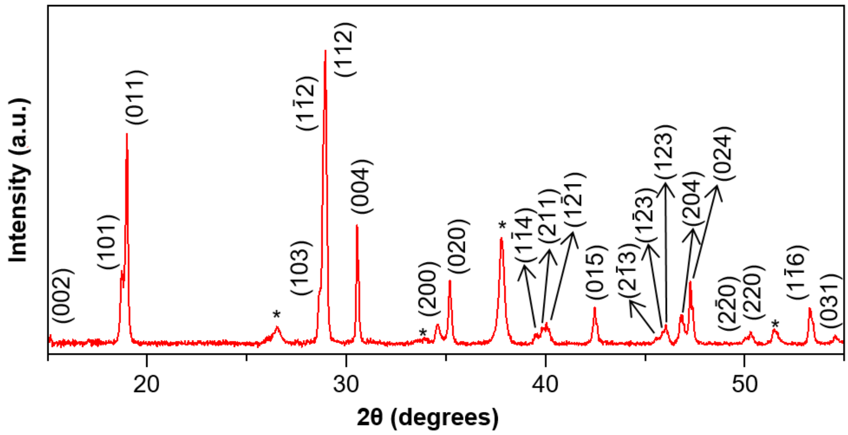

Figure S2. XRD pattern of $BiVO_4/Nb_2O_5$ showing the purity and crystallinity of $BiVO_4$ (ICSD100602). No peaks related to crystalline Nb_2O_5 were observed. Peaks from the FTO substrate are marked with asterisks (*).

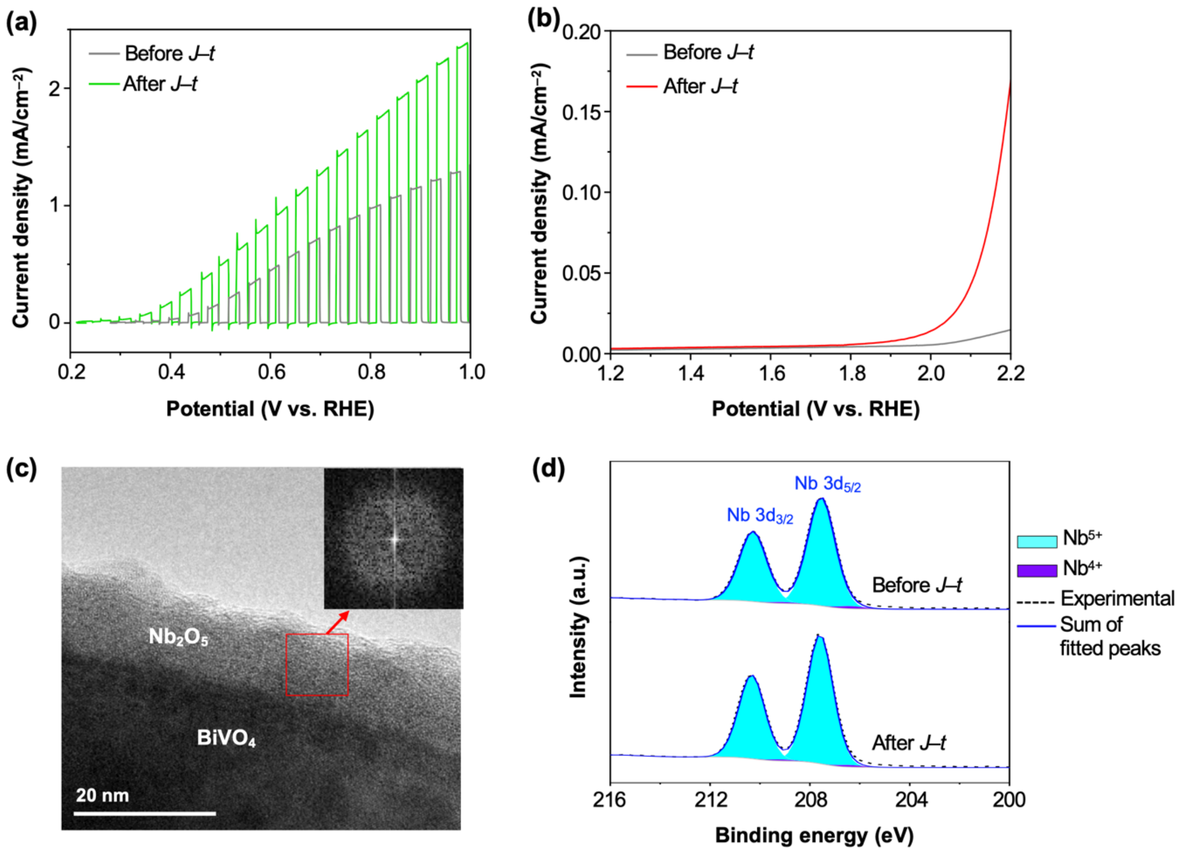

Figure S3. (a) *J–V* plots of $BiVO_4/Nb_2O_5$ for sulfite oxidation measured in 0.5 M borate buffer containing 0.4 M sulfite under AM 1.5G illumination (100 mW/cm^2) before (grey) and after (green) the activation process. (b) *J–V* plots of $BiVO_4/Nb_2O_5$ measured in 0.1 M HNO_3 comparing OER performances in the dark before (grey) and after (red) the activation process. (c) TEM image measured after the activation process showing no differences from that taken before the activation process shown in **Figure 1d**. (d) Comparison of the Nb 5*d* XPS spectra before and after the activation process. The activation process refers to the constant potential POER performed at 0.7 V vs RHE in 0.1 M HNO_3 under AM 1.5G illumination (100 mW/cm^2) for 12 h shown in **Figure 3b**.

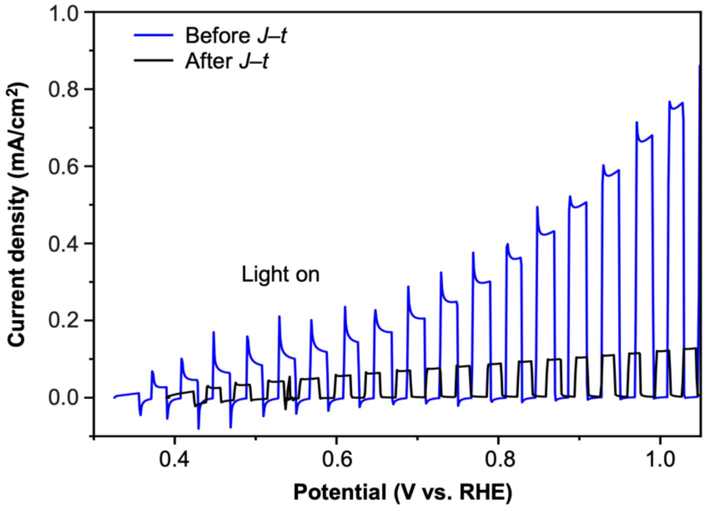

Figure S4. *J–V* plots of unprotected $BiVO_4$ measured in pH 1 HNO_3 solution under AM 1.5G illumination (100 mW/cm^2) before (blue) and after (black) the *J–t* measurement shown in **Figure 3b**.

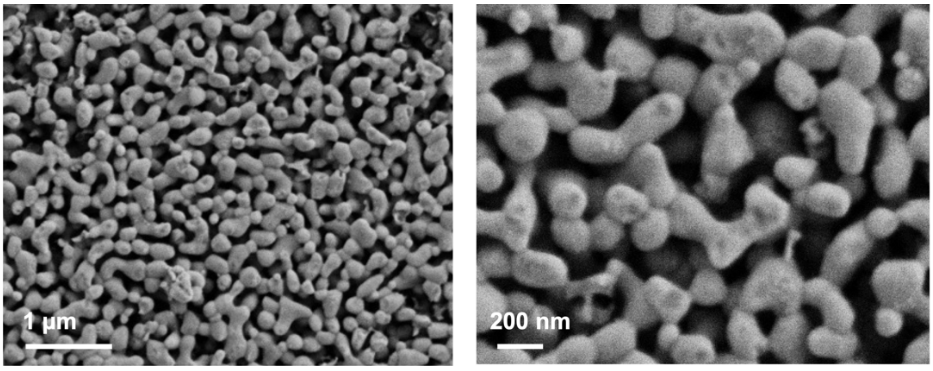

Figure S5. SEM images of unprotected $BiVO_4$ after the *J–t* measurement with Co^{2+} shown in **Figure 6b**.
